# YOLO-MARS for Infrared Target Detection: Towards near Space

**DOI:** 10.3390/s25175538

**Published:** 2025-09-05

**Authors:** Bohan Liu, Yeteng Han, Pengxi Liu, Sha Luo, Jie Li, Tao Zhang, Wennan Cui

**Affiliations:** 1 Shanghai Institute of Technical Physics, Chinese Academy of Sciences, Shanghai 200083, China; liubohan21@mails.ucas.ac.cn (B.L.);; 2 University of Chinese Academy of Sciences, Beijing 100049, China

**Keywords:** near space, YOLOv8, infrared target detection, attention mechanism, feature fusion

## Abstract

In response to problems such as large target scale variations, strong background noise, and blurred features leading by low contrast in infrared target detection in near space environments, this paper proposes an efficient detection model, YOLO-MARS, which is based on YOLOv8. The model introduces a Space-to-Depth (SPD) convolution module into the backbone section, which retains the detailed features of smaller targets by downsampling operations without information loss, alleviating the loss of the target feature caused by traditional downsampling. The Grouped Multi-Head Self-Attention (GMHSA) module is added after the backbone’s SPPF module to improve cross-scale global modeling capabilities for target area feature responses while suppressing complex thermal noise background interference. In addition, a Light Adaptive Spatial Feature Fusion (LASFF) detector head is designed to mitigate the scale sensitivity issue of infrared targets (especially smaller targets) in the feature pyramid. It uses a shared weighting mechanism to achieve adaptive fusion of multi-scale features, reducing computational complexity while improving target localization and classification accuracy. To address the extreme scarcity of near space data, we integrated 284 near space images with the HIT-UAV dataset through physical equivalence analysis (atmospheric transmittance, contrast, and signal-to-noise ratio) to construct the NS-HIT dataset. The experimental results show that ${mAP}_{@0.5}$ increases by 5.4% and the number of parameters only increase 10% using YOLO-MARS compared to YOLOv8. YOLO-MARS improves the accuracy of detection significantly while considering the requirements of model complexity, which provides an efficient and reliable solution for applications in near space infrared target detection.

## 1. Introduction

Due to developments in the international situation and related technologies, the use of stratospheric airships equipped with optoelectronic imaging systems has increased in fields such as disaster monitoring, resource exploration, and scientific research [1]. Near space generally refers to the airspace between 20 and 100 km above ground. This area is located between the highest flight altitude of civil aircraft and the lowest orbit altitude of satellites. It is also called near space, suborbital space, or the air–space transition zone. This area encompasses the atmospheric stratosphere, mesosphere, and part of the ionosphere [2]. Its good atmospheric stability, steady wind speeds and directions, minimal climate fluctuations, and abundant solar energy provide ideal conditions for the long-term residence and stable operation of airships [3].

Nowadays, near space target detection mainly relies on visible light imaging technology. However, under the conditions of weak lighting, high-altitude cloud cover, and complex backgrounds such as mountains, cities, and oceans, the detection effectiveness of visible light imaging is severely limited. Additionally, adverse weather conditions such as rain, snow, smog, and sandstorms can impair the quality of visible light images severely. This severely constrains the equipment’s detection capabilities. In contrast, infrared imaging technology has gradually become an important way of near space monitoring due to its all-weather detection capabilities, strong penetration, and long-distance detection advantages. It can work in conjunction with visible light imaging to increase stability and reliability [4,5].

However, infrared target detection in near space environments still faces numerous challenges. On the one hand, optoelectronic detectors are deployed at high altitudes. Due to limitations in device resolution and transmission capabilities, the infrared images collected typically have a low resolution, insufficient contrast, and blurred target details. On the other hand, distant targets are often small in size and dim in brightness in images, occupying only a small number of pixels. They lack obvious shape, texture, and color characteristics, leading to their being easily obscured by complex backgrounds or noise [6,7,8]. Furthermore, the scale distribution of targets in realistic scenarios is found to be uneven. The target group encompasses not only small targets but also extended targets. Substantial alterations in scale serve to compound the challenges associated with detection. The aforementioned factors impede the efficacy of conventional infrared target detection methodologies, often leading to suboptimal outcomes characterized by missed detections and the production of false positives. Concurrently, some practical applications exhibit stringent real-time requirements, thereby exacerbating the complexity of target detection. Achieving the rapid and accurate detection of multi-scale infrared targets in near space environments has become one of the key research issues in this field [9,10].

In recent times, thanks to the rapid development of computer vision technology, infrared target detection technology has made great advancements. Existing target detection algorithms fall into two categories. One category uses the traditional approach of manual feature extraction. The other category uses an end-to-end detection algorithm based on deep learning that automatically extracts target features.

Traditional algorithms primarily rely on human visual mechanisms or image feature statistics, enhancing the contrast between the background and target and thereby achieving detection. Typical methods include morphological filtering algorithms represented by Top-Hat, methods based on local contrast or sparsity modeling, and background suppression strategies using low-rank sparse decomposition. Specifically, Deshpande [11] improves the Top-Hat transformation using multi-structural element templates to enhance detection stability; Zeng [12] incorporates local contrast into the Top-Hat transformation to reduce false alarm rates. Wei [13] uses local contrast to mitigate the effects of changes in target size. Deng [14] proposes a block-based multi-scale concatenated contrast detection method that enhances target brightness while effectively suppressing the background. Kim [15] leverages the sparsity characteristics of infrared small targets and the non-local autocorrelation properties of the background to transform the small target detection problem into an image segmentation problem. Gao [16] combines local structural weights and sparse enhancement weights to achieve more precise background estimation, thereby improving detection efficiency. Dai [17] considers a density map search-based method to effectively suppress background noise.

Although they have obtained certain results in improving performance of detection, these traditional algorithms generally rely on manual experience to set thresholds and are highly sensitive to parameters. When facing rapidly changing backgrounds or complex environments, false alarm rates often increase and detection accuracy decreases. In addition, these improvements often come with an increased consumption of computing resources, making them more suitable for scenarios with relatively clean backgrounds and low real-time requirements.

By contrast, methods based on neural networks significantly improve detection and generalization capabilities by learning the high-dimensional features of targets through large-scale sample learning. These methods can be divided into two-stage detection and single-stage detection based on their structure. Two-stage algorithms such as the R-CNN series (R-CNN [18], Fast R-CNN [19], Faster R-CNN [20]) optimize separately in the object candidate stage and the classification stage. These algorithms are highly accurate but computationally expensive, making them unsuitable for resource-constrained platforms. Single-stage algorithms, such as the YOLO [21] series proposed by Redmon et al., transform the detection task into a regression problem. These algorithms offer faster inference speeds and higher deployment efficiency. YOLO has gradually become the mainstream framework for infrared target detection because of its end-to-end structure, rapid detection speed, and flexible deployment. It is also widely used in complex tasks such as infrared small targets and multi-scale targets. For example, Lin [22] proposed a GML-YOLO network that significantly reduces computational and parameter complexity while effectively improving detection accuracy through lightweight network and improved loss function. Wang [23] proposed the YOLO-HVS model, which uses a multi-scale spatially enhanced attention module to suppress background noise and enhance occluded targets, integrating local details and global context. By extending the convolution and two-step feature extraction mechanism, the efficiency of capturing multi-scale contextual information has been significantly improved, achieving efficient real-time infrared small target detection. Huang [10] modified YOLOv3 using local contrast for infrared target detection. This model improved the ability of infrared target detection in near space environments significantly. However, the original YOLO architecture has not been optimized for infrared imaging characteristics, and it still suffers from issues such as weak response to faint targets, high-scale sensitivity, and insufficient background suppression capabilities. These limitations are particularly pronounced when processing infrared images with significant scale variations in near space environments, where performance still requires further improvement.

YOLO-MARS, a lightweight infrared target detection model, which is based on YOLOv8n, is proposed in this paper. It focuses on solving key issues such as the retention of small target details and feature adaptive fusion under scale changes to improve the practicality and robustness of the model in near space infrared images.

The first section of this paper discusses the main problems of infrared target detection in the near space environment and related research. Section 2 mainly explains the main structure of YOLO-MARS. Section 3 elaborates on the design principles of the improvement strategies and modules. Section 4 shows the dataset constructed in this paper and some experimental results including comparison experiments between YOLO-MARS and YOLOv8n, as well as ablation experiments. Finally, Section 5 summarizes the work conducted in the article.

The main contributions of the work in this article are summarized in brief as follows:

(1) It improves traditional downsampling by introducing the SPD-Conv [24] module to reconstruct the backbone part, reducing information loss of smaller targets in deep features.
(2) A Grouped Multi-Head Self-Attention (GMHSA) mechanism is integrated into the neck to model global context across scales, enhance target responses, and suppress thermal noise, improving detection robustness.
(3) The GMHSA mechanism is integrated into the neck network, adaptively enhancing feature expression in the target area, suppressing background interference, and improving the capabilities of the model for detection.
(4) A LASFF head is designed to achieve multi-level feature adaptive fusion by a shared weight mechanism, significantly reducing computational overhead while enhancing infrared target detection accuracy.
(5) To address the scarcity of ground data in near space, a new dataset was constructed using the HIT-UAV [25] dataset, which is similar in scale and contrast to near space image targets and actual images taken by near space platforms.

## 2. The Proposed YOLO-MARS Model

**Figure 1:**
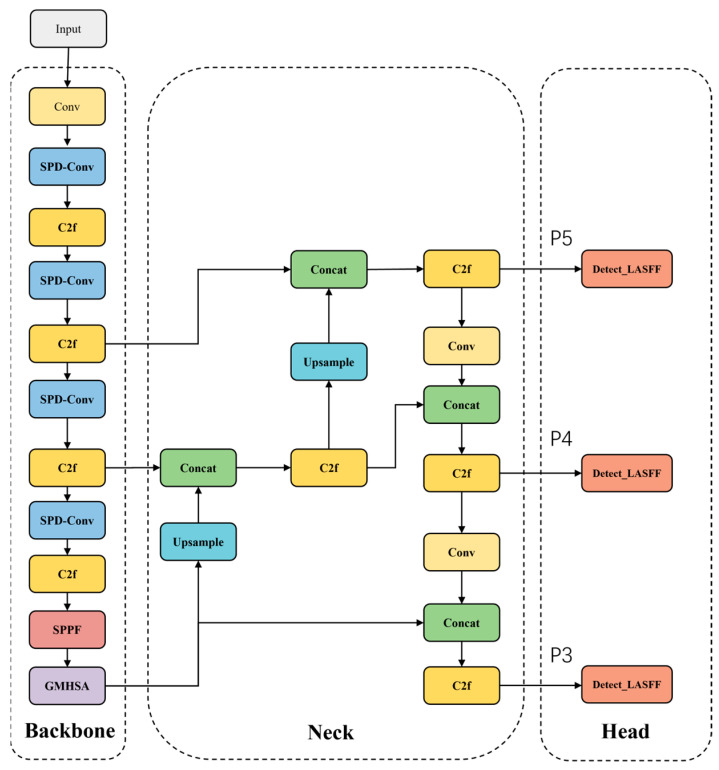
The network structure of YOLO-MARS.

To address the challenges of large target scale variations, low contrast, and strong background interference in infrared target detection in the near space environment, this paper proposes an improved model based on the YOLOv8: YOLO-MARS. The overall structure is shown in Figure 1. Similar to the structure of YOLOv8, the proposed model is mainly composed of a backbone, neck, and head. The backbone replaces the original convolution and pooling modules with the SPD-Conv [24] module. This module uses spatial feature reorganization for lossless downsampling, which preserves the edges and texture features of smaller targets. The GMHSA is added into the neck network, leveraging its global context modeling capabilities to dynamically suppress thermal noise interference while enhancing the saliency response of the target region. A lightweight adaptive detection head is designed using an ASFF [26] dynamic feature fusion strategy. This detection head achieves an efficient fusion of cross-scale features through global shared weights, reducing model complexity while enhancing the detection capability of scale-sensitive infrared targets.

## 3. YOLO-MARS Detection Model

### 3.1. Space-to-Depth Convolution (SPD-Conv)

During the downsampling process of infrared images, target details are easily lost, especially targets with small scales or blurred edge features. This paper introduces Space-to-Depth Convolution (SPD-Conv) [24] to replace the traditional convolution module for retaining more local spatial information. This module uses spatial reorganization, ensuring image size compression while preserving critical local spatial information, which enhances the ability of the model to express features of targets at different scales.

The core idea of SPD-Conv is to use Space-to-Depth operations to convert the spatial information of feature maps into channel information, aiming at reducing the loss of target feature information caused by traditional downsampling operations. Then, non-stride convolution is used to compress the number of channels. In this way, it can avoid information loss caused by stride jumps. This method effectively enhances the ability to retain details such as the edges and textures of small objects while maintaining the computational efficiency of the model. The algorithm flow is as follows.

1. Space to depth conversion

Considering that the size of input feature map is S×S×C1, and the scaling factor is scale=2, the SPD operates as follows:

(1) Feature map segmentation:

Divide the input feature map into ${scale}^{2}$ subblocks using a stride of scale. For example, when scale=2, the feature map is divided into four subblocks as follows:

(1) $$\begin{array}{cc} f_{0,0}=X[0:S:2,\;0:S:2] & f_{0,1}=X[1:S:2,\;0:S:2]\\ f_{1,0}=X[0:S:2,\;1:S:2] & f_{1,1}=X[1:S:2,\;1:S:2] \end{array}$$

Each subblock has a size of $\frac{S}{2}\times \frac{S}{2}\times C1$.

(2) Channel concatenation:

Splice the subblocks by channel dimension and obtain a new feature map $X^{\prime }$:

(2) $$X^{\prime }=Concat(f_{0,0},f_{0,1},f_{1,0},f_{1,1})$$

The size of the new feature map is $\frac{S}{2}\times \frac{S}{2}\times 4C1$.

2. Non-Strided Convolution

For the feature map $X^{\prime }$, use convolution (for example, 3 × 3, stride = 1) to compress the number of channels as follows:

(3) $$X^{\prime \prime }={Conv}_{3\times 3,stride=1}(X^{\prime })$$

This operation results in an output feature map of size $\frac{S}{2}\times \frac{S}{2}\times C2$. It reduces the number of channels from C1 to C2 through learnable parameters, achieving feature compression while preserving full spatial information.

**Figure 2:**
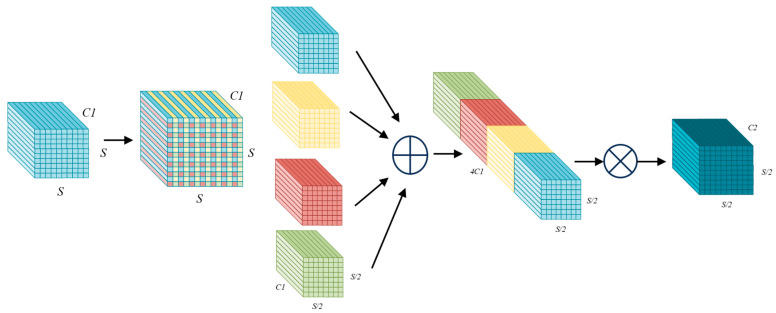
The structure of SPD-Conv when scale=2.

The size of the new feature map $X^{\prime \prime }$ is $\frac{S}{2}\times \frac{S}{2}\times C2$. This operation compresses the number of channels from 4C1 to C2 through learnable parameters, achieving feature compression while maintaining spatial information integrity. The algorithm flow is shown in Figure 2.

### 3.2. Grouped Multi-Head Self-Attention (GMHSA)

Infrared target detection faces the challenges of complex background noise and low contrast between targets and background. This paper tries to use the Multi-Head Self-Attention (MHSA) mechanism [27] to improve the model’s ability of global context modeling. However, the original MHSA is computationally intensive and does not pay enough attention to local details, which shows limitations in practical applications. We propose the Grouped Multi-Head Self-Attention (GMHSA) mechanism. This mechanism balances global perception and local modeling by grouping channels, thereby reducing computational complexity and improving the ability of recognizing targets with multiple scales in infrared images. The core of GMHSA is to divide the input features into several groups according to the channel dimension, and then perform multi-head attention modeling in each group. This structure not only retains the ability of the multi-head mechanism to model different feature subspaces, but also improves the accuracy and computational efficiency of local detail modeling by grouping strategies.

Specifically, considering the input feature map $X\in R^{H\times W\times C}$, the GMHSA process is as follows.

1. Feature Projection

Flatten the input feature map $X\in R^{H\times W\times C}$ into a sequence form, with a length of L= H·W. Divide the feature map into g groups according to the channel dimension (in this paper, g=2). The scale of each group is $R^{L\times C/g}$. Then each group generates Query(Q), Key(K), and Value(V) matrices through three independent linear transformations occur as follows:

(4) $$Q=XW^{Q},K=XW^{K},V=XW^{V}$$

where $W^{Q}{,W}^{K},W^{V}$ are learnable weight matrices.

2. Multi-head segmentation and attention calculation

Divide the Q, K, and V of each group into h submatrices according to the number of heads h (in this paper, h=4):

(5) $$Q=\left[Q_{1},Q_{2},\ldots ,Q_{h}\right],K=\left[K_{1},K_{2},\ldots ,K_{h}\right],V\;=\;[V_{1},V_{2},\ldots ,V_{h}]$$

where the size of each submatrix is $R^{L\times d_{k}}$, and $d_{k}=\;C\;/\;(g\cdot h)$.

Each head independently calculates the result of self-attention as follows:

(6) $${head}_{i}=Softmax\left(\frac{Q_{i}K_{i}^{T}}{\sqrt{d_{k}}}\right)V_{i}\;(i=1,2,\ldots ,h)$$

3. Concatenate and output fusion

**Figure 3:**
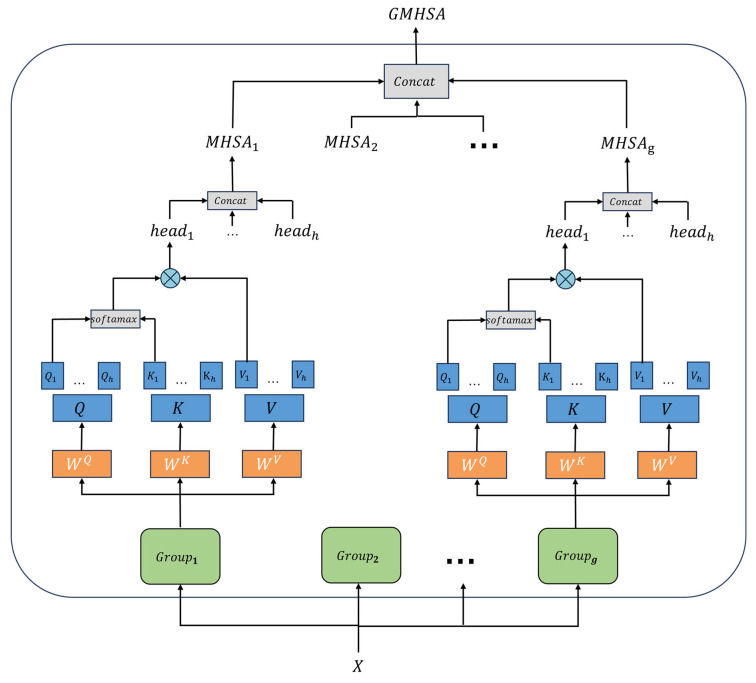
The structure of the GMHSA module.

Concatenate the outputs of attention heads within each group in the channel dimension. Then, fuse and concatenate the outputs of each group. And, finally, restore the feature dimension through linear mapping as follows:

(7) $$GMHSA\;=\;{Concat}_{j=1}^{g}({Concat}_{i=1}^{h}\left({head}_{i}^{j}\right))W^{O}$$

where $W^{O}$ is the output projection matrix. The algorithm flow is shown in Figure 3.

### 3.3. Lightweight Adaptive Spatial Feature Fusion Head (LASFF Head)

The detection head of YOLOv8 uses fixed weights to fuse multi-scale features. However, in near space infrared images, factors such as detection distance and size significantly affect ground targets, leading to significant scale differences. This situation causes existing detection heads to perform poorly in target detection tasks with multiple scales.

To improve the ability of the model to adapt to scale changes, Liu et al. [26] proposed a new feature pyramid fusion algorithm called ASFF (Adaptive Spatial Feature Fusion). Unlike the fixed-weighting method used in traditional FPN, ASFF can adaptively learn spatial weights from the given image, dynamically adjusting the fusion weights of features at different scales. This algorithm effectively alleviates the conflict between multi-scale features in the feature pyramid, which retains more useful features and enhances the consistency of multi-scale detection.

**Figure 4:**
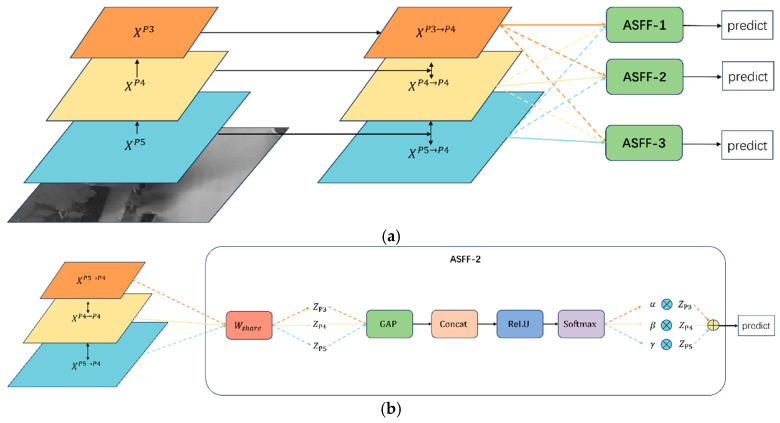
Structure of the LASFF detection head. (**a**) Structure of the LASFF; (**b**) Structure of the ASFF-2 Detection Head.

Although the original ASFF improved the model detection performance, it also led to large computational and parameter quantities, which are not suitable for application in near space embedded devices. Therefore, we designed a lightweight adaptive spatial feature fusion detection head (LASFF head). Based on the adaptive principles of ASFF, this paper introduced a shared weight mechanism to significantly reduce the consumption of calculation and the number of parameters while maintaining a good capacity of detection. Figure 4 shows the structure of the detection head. The specific implementation process for LASFF heads is as follows:

1. Feature alignment

The feature pyramid of YOLOv8 consists of three layers (P3, P4, P5). When P4 is the target layer, the feature maps of P3 and P5 need to be adjusted to the same resolution as P4.

For layer P3: Since layer P3 has a lower resolution than layer P4, upsampling is used to align the sizes of P3 to P4.

(8) $$X^{P3\to P4}=Upsample(X^{P3})$$

For layer P5: Since layer P5 has a higher resolution than layer P4, convolution downsampling is used to align the sizes of P5 to P4.

(9) $$X^{P5\to P4}=Conv(X^{P5})$$

2. Shared weight generation

Use shared convolutions to align feature maps across channels as follows:

(10) $$Z_{k}=W_{share}\;*\;X^{k\to P4}\;\;\;\;k\in \{P3,P4,P5\}$$

Unlike the original ASFF, which generates independent weights for each scale, LASFF detection heads use a unified global weight generation module.

Global average pooling for global feature extraction is calculated as follows:

(11) $$g_{k}=\;GAP(Z_{k})$$

Feature concatenation and weight generation are determined as follows:

(12) $$g=\;Concat\left(g_{p3},g_{p4},g_{p5}\right)$$

(13) $$\left|\alpha ,\beta ,\gamma \right|=Softmax(W_{2}\cdot ReLU\left(W_{1}g+b_{1}\right)+b_{2},\;\dim=1)$$

where α is the weight of P3, β is the weight of P4, and γ is the weight of P5, satisfying α+β+γ=1.

3. Feature fusion


(14) $$Y^{P4}=\alpha \cdot Z_{P3}+\beta \cdot Z_{P4}+\gamma \cdot Z_{P5}$$

The fused feature map $Y^{P4}$ is then used for target classification and bounding box regression.

By adding the shared weight mechanism, LASFF significantly reduces the number of parameters and consumption of computation while retaining its advantage in dynamic fusion. This effectively improves the robustness of the detection head in detecting multi-scale targets in near space infrared images and provides good real-time performance and deployment efficiency, making it applicable for detection devices with limited computing resources.

## 4. Experiments and Results

### 4.1. Dataset

Due to the limitations of observation environments and imaging conditions in near space infrared imaging, publicly available high-quality datasets remain extremely scarce.

To construct training samples that better reflect practical application scenarios, this study collected 284 real-world infrared images during a near space experimental mission conducted by the authors.

This article analyzes these images, along with the publicly available HIT-UAV dataset [25].

The essence of infrared imaging is the process in which target radiation is attenuated by the atmosphere and received by sensors. Its mathematical model can generally be expressed as follows:

(15) $$I_{sensor}=\;\tau \cdot \epsilon \cdot B\left(T\right)+\left(1-\tau \right)\cdot I_{atm}+N$$

$I_{sensor}$ represents the radiation intensity received by the sensor, τ is the atmospheric transmittance, ϵ is the target emissivity, $B\left(T\right)$ is the blackbody radiation intensity, $I_{atm}$ is the atmospheric path radiation, and N is the system noise. The key parameter to pay attention to during the propagation of infrared radiation is the atmospheric transmittance τ.

**Figure 5:**
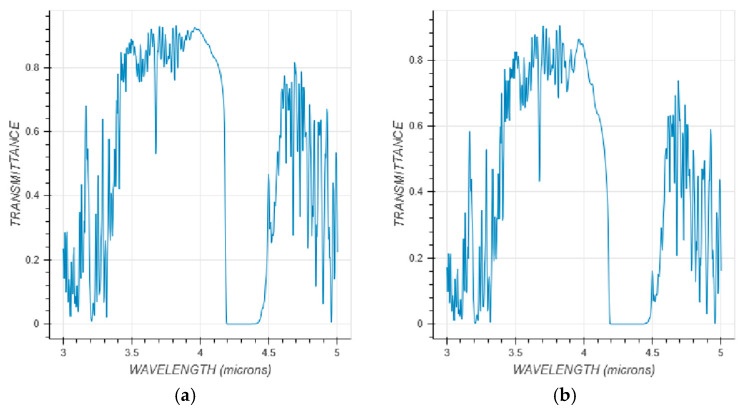
Comparison of atmospheric transmittance at different altitudes. (**a**) Infrared atmospheric transmittance (2 km); (**b**) Infrared atmospheric transmittance (20 km).

MODTRAN is a widely used software developed in the United States for calculating atmospheric transmittance. As shown in Figure 5, using MODTRAN analysis under the same basic conditions, the atmospheric transmittance in the mid-wave infrared band is very close between 2 km (UAV working altitude) and 20 km (near space altitude). During the experiment, the authors obtained the near space images under better environmental conditions, such as clear and cloudless weather, thinner air on the ground in high-altitude areas, and higher visibility.

In addition, the average contrast (AC) and signal-to-noise ratio (SNR) between the target and background in the image were also calculated. The results are as follows in Table 1:

Although both indicators of the actual captured images have slightly decreased, in situations where near space data is extremely scarce, this equivalent application is acceptable.

Based on the above analysis, these images are integrated with the publicly available HIT-UAV dataset, which has similar features in target contrast, signal-to-noise ratio, and some background, to build a near space infrared target detection dataset named NS-HIT.

The NS-HIT dataset includes five typical categories of ground targets and more closely matches the actual distribution and visual characteristics of targets observed from near space platforms, which includes 2217 training images, 642 test images, and 323 validation images.

Details of the dataset samples are presented in Table 2.

**Figure 6:**
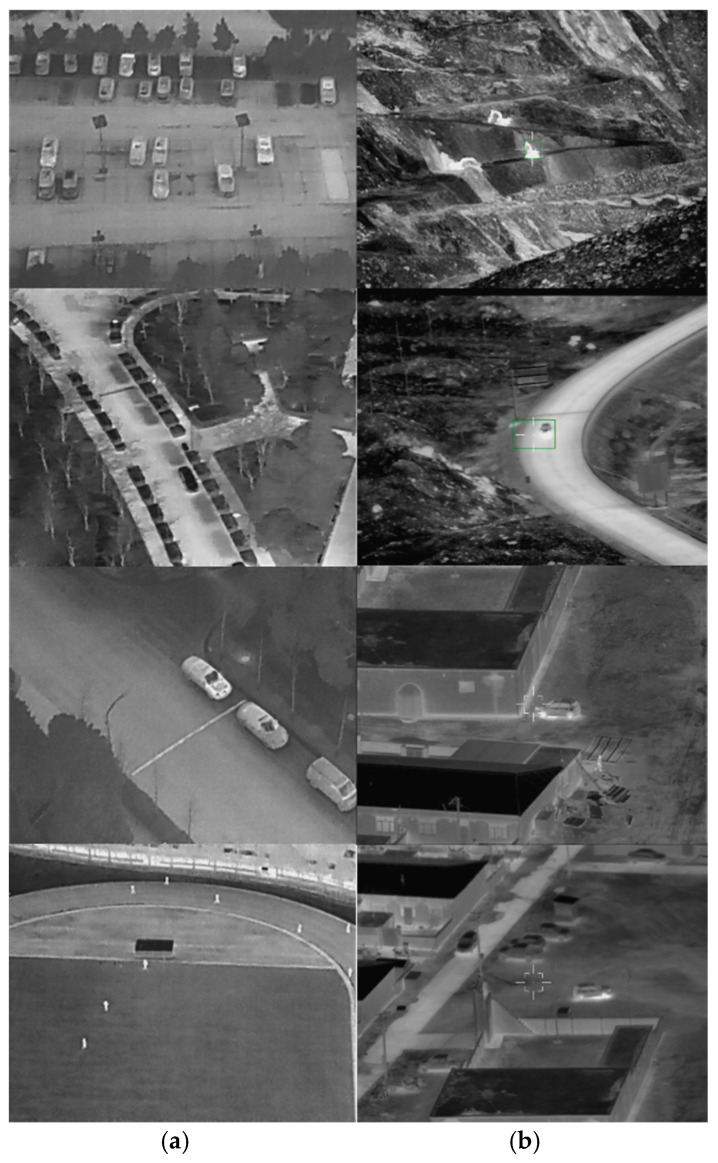
Comparison of dataset images (**a**) sample images from the HIT-UAV dataset; (**b**) actual images captured by our near space device.

Figure 6a shows some sample images from the HIT-UAV dataset and Figure 6b shows actual images captured by our near space device. The two sets of images share certain similarities in terms of targets and size. We combine these data for the training and evaluation of the near space infrared target detection model. Under poor conditions lacking data in near space, it can improve the adaptability and generalization performance of this model.

### 4.2. Experimental Indicators

For the purpose of evaluating the capability of YOLO-MARS in infrared target detection tasks, this article uses the following two indicators:

(1) Detection Accuracy:

- ${mAP}_{@0.5}$: The mean Average Precision (mAP) is calculated using an IoU threshold of 0.5, reflecting the model’s capability in accurately locating targets.
- ${mAP}_{@0.5:0.95}$: The mean Average Precision is calculated using IoU thresholds ranging from 0.5 to 0.95, with a step size of 0.05. This provides a more comprehensive assessment of the model’s performance in localization and classification.

(2) Model Complexity:

- Parameters: The total number of learnable parameters in the model.
- FLOPs: The number of floating-point operations required for a single forward pass, indicating computational complexity.
- Model Size: The disk storage size of the model file, which affects its suitability for deployment on edge or embedded devices.

The above indicators comprehensively reflect model performance in both detection accuracy and resource consumption.

For detection accuracy evaluation, the Average Precision (AP) for each category is defined as the integral of precision over the recall range as follows:

(16) $${AP}_{C}=\;\int_{0}^{1}{Precision}_{c}(r)dr$$

(17) $$Precision=\frac{TP}{TP+FP}$$

(18) $$mAP=\frac{1}{N}\sum_{c=1}^{N}{AP}_{C}$$

where TP is the number of correctly identified positive samples, and FP is the number of negative samples incorrectly identified as positive.

### 4.3. Experiments and Results Analysis

#### 4.3.1. Experimental Environment

The experiments ran on a Linux operating system, with an NVIDIA GeForce RTX 3090 GPU (24 GB) and a 20-core AMD EPYC 7642 processor. The framework used was PyTorch 2.1.2, and was accelerated by CUDA 11.8. The input images were resized to 640 × 640. Stochastic Gradient Descent (SGD) was used as the optimization function, and the mixed precision training amp was turned off.

The model used in the experiment is based on the YOLOv8n (all experiments in the following are conducted based on this version by default). The epochs were 300. The batch size and the number of data loading threads were set to 16 and 4, and the initial learning rate was 0.001.

#### 4.3.2. Validation of Space-to-Depth Convolution Module

In order to verify the improvement of the SPD-Conv module in infrared target detection, this paper replaces part of the convolution layers in the original YOLOv8n with the SPD-Conv module. It can be seen from the contents in Table 3 that the improved YOLOv8n-SPDConv demonstrates better performance than the original version. The ${mAP}_{@0.5}$ improved by 1.8%. At the same time, the value of the parameters and size both decreased.

The improvement proves that SPD-Conv can downsample without information loss by spatial reorganization, fully retaining the edge and texture details of smaller targets. It can effectively alleviate the information loss caused by traditional convolution, which improves detection accuracy and model efficiency.

#### 4.3.3. Comparative Experiments on Attention Mechanisms

To make a comparison between the GMHSA mechanism discussed in this paper and other attention mechanisms, we used the common attention mechanism modules SimAM [28], ACmix [29], Biformer [30], CoTAttention [31], and RFEM [32], which were added into the same position of YOLOv8n. Specifically, it is after the SPPF. The results are recorded in Table 4.

It can be seen that the GMHSA mechanism improved ${mAP}_{@0.5}$ and ${mAP}_{@0.5:0.95}$ significantly compared with the attention mechanisms which are generally common. Even though the Biformer module has a higher ${mAP}_{@0.5}$, it also brings significantly higher model complexity (with a significant increase in parameter count and FLOPs). GMHSA balances detection accuracy and model efficiency, showing better practicality and robustness in near space infrared target detection tasks.

#### 4.3.4. Validation of the Lightweight Adaptive Detection Head

To validate the improvements in model detection performance and model complexity achieved by the LASFF detection head proposed in this paper, we conducted a comparative experiment between the original YOLOv8n and the improved model with the LASFF detection head. The results are shown in Table 5.

Table 5 shows that the detection accuracy of the YOLOv8n-LASFF model is better than YOLOv8n. Specifically, ${mAP}_{@0.5}$ is improved by 2.7%, while keeping a low number of parameters and low consumption for computation. This suggests that LASFF can effectively alleviate the scale inconsistency problem of infrared targets in the pyramid feature, enhancing the adaptability of feature fusion for multi scales, which improves the ability of the model for the classification and localization of multi-scale targets. In addition, since this module uses shared weights and structure simplification strategies, it meets the requirements for model deployment and real-time application on embedded near space platforms.

#### 4.3.5. Ablation Experiments

In order to better demonstrate the effect of several improvements in YOLO-MARS on the performance of detection, this paper performed ablation experiments on the several improvements of this model based on YOLOv8n. The results of the ablation experiments on the NS-HIT dataset are shown in Table 6, which shows the improvements gradually step by step. The symbol √ indicates that the model used in this experiment includes this module.

Table 6 shows that each proposed improvement contributes to the accuracy of detection in this model. Compared with YOLOv8n, the YOLO-MARS improved ${mAP}_{@0.5}$ by 5.4% and ${mAP}_{@0.5:0.95}$ by 3.8% on the NS-HIT dataset, validating the effectiveness and synergistic benefits of each module.

The structural complexity has increased, with the number of parameters and the complexity of the model increasing by approximately 10% and 17%, and the increase in FLOPs has led to a slight decrease in inference frame rate. This is reasonable and acceptable in exchange for the improvement in detection accuracy. The improved model still maintains a light weight.

**Figure 7:**
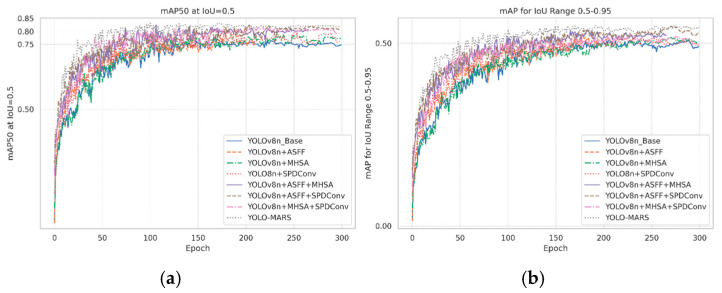
Comparison curves of the accuracy in model training. (**a**) The Learning Curve of ${mAP}_{@0.5}$; (**b**) The Learning Curve of ${mAP}_{@0.5:0.95}$.

To further illustrate the impact of the proposed improvements on training performance, Figure 7 shows the accuracy change curves of YOLO-MARS and its various improvements during the training process. From the early stages of training, it can be observed that YOLO-MARS significantly outperforms YOLOv8n. It continues to maintain a higher level of accuracy in the later stages, indicating that the proposed modules effectively enhance the model’s feature representation capabilities and convergence stability.

### 4.4. Comparative Experiments

In order to prove the excellence of the proposed YOLO-MARS model in infrared target detection in near space, comparative experiments were made between this model and current mainstream target detection algorithms, including the YOLO series, Faster R-CNN (ResNet-101), and Sparse R-CNN (ResNet-50) [33] All models were trained on the NS-HIT dataset. Training parameters and hyperparameters were kept consistent to ensure fairness and consistency in comparison.

From the comparison results in Table 7, it can be concluded that YOLO-MARS is significantly better in detection accuracy, outperforming other models in both ${mAP}_{@0.5}$ and ${mAP}_{@0.5:0.95}$. At the same time, YOLO-MARS controls the growth of parameter numbers and model complexity, which improves accuracy without significantly increasing computational overhead. These results further validate the practical value of YOLO-MARS in near space infrared target detection.

### 4.5. Visualization and Analysis of Detection Results

**Figure 8:**
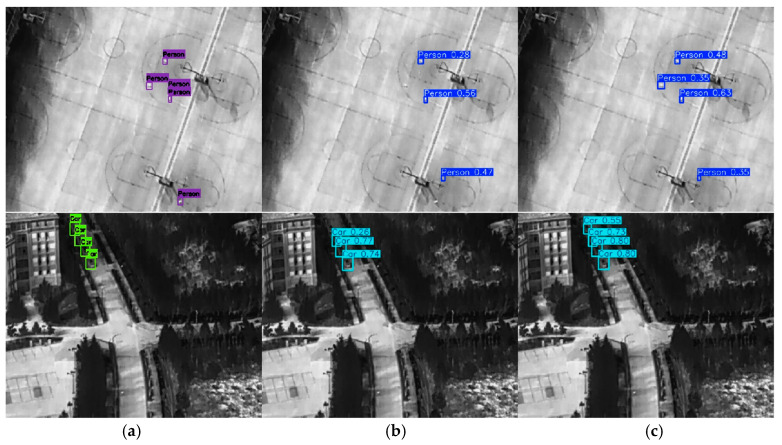
Comparison of detection results between YOLOv8 and YOLO-MARS: (**a**) Original Annotations; (**b**) Detection results of YOLOv8; (**c**) Detection results of YOLO-MARS.

To further illustrate the superiority of the proposed YOLO-MARS model, Figure 8 shows a comparison of the detection results between the original YOLOv8 model and YOLO-MARS on pictures from the test set.

Figure 8 shows that the YOLO-MARS can successfully detect low-contrast, small-scale targets that the original model failed to identify or misidentified, demonstrating stronger robustness and detection capabilities. This verified that the improvements of YOLO-MARS proposed in this paper have obvious advantages in dealing with the common issues of low resolution and targets with multiple scales in near space infrared images, which shows great potential for application.

## 5. Conclusions

To address issues such as the low resolution of images, significant target scale variation, and limited feature representation in near space infrared target detection, this paper proposes a high-precision detection model, YOLO-MARS, based on YOLOv8. The proposed method reconstructs the downsampling layers of the backbone section using the SPD-Conv module, enabling downsampling without losing spatial information and preserving the edge and texture features of smaller targets. By combining the GMHSA mechanism into the neck section, the model enhances the response of the target area while suppressing interference from complex thermal noise in infrared images, which improves its ability to distinguish key areas. A LASFF detector replaced the original detector. It dynamically fuses the multi-scale features of the images, improving the ability of the proposed model to locate and classify targets which are sensitive to scale changes.

On the NS-HIT dataset, YOLO-MARS outperforms the baseline model by 5.4% in ${mAP}_{@0.5}$ and by 3.8% in ${mAP}_{@0.5:0.95}$. In addition, thanks to its lightweight structural design, YOLO-MARS only increased the parameter count by approximately 10% and model size by 17%, maintaining a high runtime efficiency while improving detection accuracy.

In summary, YOLO-MARS demonstrates excellent detection accuracy and deployment efficiency in near space infrared target detection tasks, which provides an efficient and robust solution for resource-constrained embedded systems and edge computing platforms. Future work will extend this approach to newer architectures like YOLOv12, exploring the better performance of the model.

## Figures and Tables

**Table 1:** Comparison between HIT-UAV and data from near space.

Parameter	HIT-UAV	Data from Near Space
AC	0.094	0.0902
SNR	0.72	0.67

**Table 2:** Labels and targets in NS-HIT.

Labels	Train Sets Amount	Test Sets Amount	Val Sets Amount
Person	8612	2641	1182
Car	5412	1369	735
Bicycle	3630	796	554
OtherVehicle	132	42	15
Dontcare	119	32	7

**Table 3:** Comparison of reconfigured model using SPD-Conv.

	YOLOv8n	YOLOv8n-SPDConv
${mAP}_{@0.5}$	0.778610	0.78736
${mAP}_{@0.5:0.95}$	0.500080	0.51155
Params (M)	3.01	2.81
FLOPS (G)	8.2	2.3
$Model\;\left(MB\right)$	5.97	5.57

**Table 4:** Comparison results of different attention mechanisms.

	mAP_@0.5_	mAP_@0.5:0.95_	Params (M)	FLOPS (G)	Model (MB)
YOLOv8n (Baseline)	0.778	0.500	3.01	8.2	5.97
GMHSA (Ours)	0.796	0.513	3.24	8.5	6.42
SimAM	0.781	0.503	3.01	8.2	5.98
ACmix	0.778	0.501	3.23	6.9	6.38
Biformer	0.798	0.512	3.88	9.4	9.24
CoTAttention	0.784	0.509	3.73	9.1	7.38
RFEM	0.773	0.495	3.23	6.8	6.38

**Table 5:** Comparison of reconfigured model using LASFF.

	YOLOv8n	YOLOv8n-LASFF
${mAP}_{@0.5}$	0.778610	0.80604
${mAP}_{@0.5:0.95}$	0.500080	0.51640
Params (M)	3.01	3.46
FLOPS (G)	8.2	9.0
$Model\;\left(MB\right)$	5.97	6.98

**Table 6:** Results of ablation experiments on NS-HIT dataset.

Baseline	GMHSA	LASFF	SPD-	mAP_@0.5_	mAP_@0.5:0.95_	P (%)	R (%)	Params (M)	FLOPSG (G)	FPS	Mode (MB)
√				0.77861	0.50008	0.788	0.675	3.01	8.2	66.44	5.97
√	√			0.79637	0.51331	0.883	0.743	3.24	8.5	56.34	6.42
√		√		0.80604	0.51640	0.820	0.758	3.46	9.0	56.75	6.98
√			√	0.78736	0.51155	0.806	0.708	2.81	2.3	63.23	5.57
√	√	√		0.82537	0.51906	0.915	0.762	3.71	9.2	50.91	7.72
√	√		√	0.81623	0.51534	0.889	0.747	3.02	7.9	61.53	6.01
√		√	√	0.82425	0.52627	0.821	0.787	3.25	8.4	57.45	6.84
√	√	√	√	0.83273	0.53844	0.871	0.778	3.32	8.7	58.34	7.03

√ indicates that the model used in this experiment includes this module.

**Table 7:** Comparison of training results of different models on NS-HIT.

Model Name	mAP_@0.5_	mAP_@0.5:0.95_	Params (M)	FLOPS (G)	Model (MB)
YOLOv3t	0.76728	0.4318	12.1	19.0	23.27
YOLOV5n	0.78611	0.49068	2/51	7.2	5.05
YOLOv6	0.74769	0.44364	4.24	11.9	8.31
YOLOv8n	0.77861	0.50008	3.01	8.2	5.97
YOLOv9t	0.78783	0.49914	2.01	7.9	4.45
YOLOv10n	0.7893	0.48424	2.71	8.4	5.51
YOLOv11	0.79506	0.48462	2.59	6.4	5.24
YOLOv12	0.77939	0.48093	2.57	6.5	5.29
FasterRCNN (R101)	0.641	0.389	45	——	253
Sparse RCNN (R50)	0.469	0.253	32	——	453.9
YOLO-MARS	0.83273	0.53844	3.32	8.7	7.03

## Data Availability

The HIT-UAV data were downloaded from https://www.nature.com/articles/s41597-023-02066-6 (accessed on 10 February 2025). The data that support the findings of this study are available from the corresponding author upon reasonable request.

## References

[1] Ruan W. (2025). A Brief Discussion on the Development Status and Strategic Significance of Stratospheric Airships. J. East China Technol..

[2] Lu X., Li H., Li X., Te R., Geng J., He X. (2024). Near space development and utilization. J. China Aerospace..

[3] Xiao Z., Ji R., Chen A., Qi Z., Chi F., Li L. (2024). Development Status and Prospects of Low Speed Aircraft in Near Space. J. China Aerospace..

[4] Lou Y., Zhang Y., Ming D., Tian J. (2016). Research on multispectral detection technology of near space target. J. Ship Electron. Eng..

[5] Li G., Tang P., Kang G., Li Z. (2016). Design of simulation experiments for detection of near-space based targets. J. Exp. Sci. Technol..

[6] Sun R. (2012). Analysis of the role of infrared detection in early warning aircraft. J. Laser Infrared..

[7] Nie W., Luo S., Feng S., Zhuang F. (2012). Analysis of key technology and development trend of near-space vehicle. J. Natl. Univ. Def. Technol..

[8] Li F., Wang Z., Zhou F., An C., Cao G. (2014). Key technology of optoelectronic reconnaissance system for stratospheric airship platform. J. Adv. Laser Optoelectron..

[9] Han J. (2015). Research on Infrared Target Detection Method for Space-Based Platforms in Near Space. Master’s Thesis.

[10] Huang X. (2021). Research on Infrared Imaging and Detection Technology of Proximity Space Platform. Master’s Thesis.

[11] Deshpande S.D., Er M.H., Venkateswarlu R., Chan P. (1999). Max-mean and max-median filters for detection of small targets. Signal Data Process. Small Targets.

[12] Zeng M., Li J., Peng Z. (2006). The design of top-hat morphological filter and application to infrared target detection. J. Infrared Phys. Technol..

[13] Wei Y., You X., Li H. (2016). Multiscale patch-based contrast measure for small infrared target detection. Pattern Recognit..

[14] Deng H., Sun X., Liu M., Ye C., Zhou X. (2016). Infrared small-target detection using multiscale gray difference weighted image entropy. IEEE Trans. Aerosp. Electron. Syst..

[15] Kim S., Lee J. (2012). Scale invariant small target detection by optimizing signal-to-clutter ratio in heterogeneous background for infrared search and track. Pattern Recognit..

[16] Gao C., Meng D., Yang Y., Wang Y., Zhou X., Hauptmann A.G. (2013). Infrared Patch-Image Model for Small Target Detection in a Single Image. IEEE Trans. Image Process..

[17] Dai Y., Wu Y. (2017). Reweighted Infrared Patch-Tensor Model with Both Nonlocal and Local Priors for Single-Frame Small Target Detection. IEEE J. Sel. Top. Appl. Earth Obs. Remote Sens..

[18] Girshick R., Donahue J., Darrell T., Malik J. Rich feature hierarchies for accurate object detection and semantic segmentation. Proceedings of the IEEE Conference on Computer Vision and Pattern Recognition (CVPR 2014).

[19] Girshick R. Fast R-CNN. Proceedings of the IEEE International Conference on Computer Vision (CVPR 2015).

[20] Ren S., He K., Girshick R., Sun J. (2015). Faster R-CNN: Towards real-time object detection with region proposal networks. Adv. Neural Inf. Process. Syst..

[21] Redmon J., Divvala S., Girshick R., Farhadi A. You only look once: Unified, real-time object detection. Proceedings of the IEEE Conference on Computer Vision and Pattern Recognition (CVPR 2016).

[22] Jiang L., Shen Y., Da M., Hu J., Zhang Z. (2025). GML-YOLO: A lightweight infrared small target detection algorithm. Meas. Sci. Technol..

[23] Wang X., Sheng Y., Hao Q., Hou H., Nie S. (2025). YOLO-HVS: Infrared Small Target Detection Inspired by the Human Visual System. Biomimetics.

[24] Sunkara R., Luo T. (2023). No more strided convolutions or pooling: A new CNN building block for low-resolution images and small objects. Joint European Conference on Machine Learning and Knowledge Discovery in Databases, Proceedings of the European Conference on Machine Learning and Principles and Practice of Knowledge Discovery in Databases, Grenoble, France, 19–23 September 2022.

[25] Suo J., Wang T., Zhang X., Chen H., Zhou W., Shi W. (2023). HIT-UAV: A high-altitude infrared thermal dataset for Unmanned Aerial Vehicle-based object detection. Sci. Data.

[26] Liu S., Huang D., Wang Y. (2019). Learning spatial fusion for single-shot object detection. arXiv.

[27] Vaswani A., Shazeer N., Parmar N., Uszkoreit J., Jones L., Gomez A.N., Kaiser Ł., Polosukhin I. Attention is all you need. Proceedings of the 31st Conference on Neural Information Processing Systems (NIPS 2017).

[28] Qin X., Li N., Weng C., Su D., Li M. Simple attention module based speaker verification with iterative noisy label detection. Proceedings of the ICASSP 2022-2022 IEEE International Conference on Acoustics, Speech and Signal Processing (ICASSP).

[29] Pan X., Ge C., Lu R., Song S., Chen G., Huang Z., Huang G. On the integration of self-attention and convolution. Proceedings of the IEEE/CVF Conference on Computer Vision and Pattern Recognition (CVPR 2022).

[30] Zhu L., Wang X., Ke Z., Zhang W., Lau R. Biformer: Vision transformer with bi-level routing attention. Proceedings of the IEEE/CVF Conference on Computer Vi-sion and Pattern Recognition (CVPR 2023).

[31] Li Y., Yao T., Pan Y., Mei T. (2022). Contextual Transformer Networks for Visual Recognition. IEEE Trans. Pattern Anal. Mach. Intell..

[32] Yu Z., Huang H., Chen W., Su Y., Liu Y., Wang X. (2024). YOLO-FaceV2: A scale and occlusion aware face detector. Pattern Recognit..

[33] Sun P., Zhang R., Jiang Y., Kong T., Xu C., Zhan W., Tomizuka M., Li L., Yuan Z., Wang C. Sparse R-CNN: End-to-end object detection with learnable proposals. Proceedings of the IEEE/CVF Conference on Computer Vision and Pattern Recognition (CVPR 2021).

